# Bacterial adhesion on conventional and self-ligating metallic brackets after surface treatment with plasma-polymerized hexamethyldisiloxane

**DOI:** 10.1590/2177-6709.22.4.077-085.oar

**Published:** 2017

**Authors:** Rogerio Amaral Tupinambá, Cristiane Aparecida de Assis Claro, Cristiane Aparecida Pereira, Celestino José Prudente Nobrega, Ana Paula Rosifini Alves Claro

**Affiliations:** 1 Universidade Estadual Paulista, Faculdade de Engenharia, Departamento de Materiais (Guaratinguetá/SP, Brasil).; 2 Universidade de Taubaté, Faculdade de Odontologia, Departamento de Ortodontia (Taubaté/SP, Brasil).; 3 Universidade Estadual Paulista, Faculdade de Odontologia, Departamento de Microbiologia e Imunologia, Instituto de Ciência e Tecnologia (São José dos Campos/SP, Brasil).; 4 New York University, College of Dentistry, Linhart Continuing Education Program (New York/NY, EUA).

**Keywords:** Orthodontic brackets, Bacterial adhesion, Hexamethyldisiloxane.

## Abstract

**Introduction:**

Plasma-polymerized film deposition was created to modify metallic orthodontic brackets surface properties in order to inhibit bacterial adhesion.

**Methods:**

Hexamethyldisiloxane (HMDSO) polymer films were deposited on conventional (n = 10) and self-ligating (n = 10) stainless steel orthodontic brackets using the Plasma-Enhanced Chemical Vapor Deposition (PECVD) radio frequency technique. The samples were divided into two groups according to the kind of bracket and two subgroups after surface treatment. Scanning Electron Microscopy (SEM) analysis was performed to assess the presence of bacterial adhesion over samples surfaces (slot and wings region) and film layer integrity. Surface roughness was assessed by Confocal Interferometry (CI) and surface wettability, by goniometry. For bacterial adhesion analysis, samples were exposed for 72 hours to a *Streptococcus mutans* solution for biofilm formation. The values obtained for surface roughness were analyzed using the Mann-Whitney test while biofilm adhesion were assessed by Kruskal-Wallis and SNK test.

**Results:**

Significant statistical differences (*p*< 0.05) for surface roughness and bacterial adhesion reduction were observed on conventional brackets after surface treatment and between conventional and self-ligating brackets; no significant statistical differences were observed between self-ligating groups (*p*> 0.05).

**Conclusion:**

Plasma-polymerized film deposition was only effective on reducing surface roughness and bacterial adhesion in conventional brackets. It was also noted that conventional brackets showed lower biofilm adhesion than self-ligating brackets despite the absence of film.

## RESUMO

**Introdução:**

a deposição de filme de polímero a plasma foi criada para modificar as propriedades de superfície dos braquetes ortodônticos metálicos, com o intuito de inibir a adesão bacteriana.

**Métodos:**

filmes finos de polímero de hexametildisiloxano (HMDSO) foram depositados em braquetes ortodônticos de aço inoxidável convencionais (n = 10) e autoligáveis (n = 10), utilizando a técnica de radiofrequência PECVD (*Plasma-Enhanced Chemical Vapor Deposition*). As amostras foram divididas em dois grupos, de acordo com o tipo de braquete, e dois subgrupos após o tratamento de superfície. A microscopia eletrônica de varredura (MEV) foi realizada para avaliar a presença de adesão bacteriana sobre as superfícies das amostras (região de ranhura horizontal e aletas) e a integridade da camada de filme. A Interferometria Confocal (CI) avaliou a rugosidade, e a molhabilidade superficial foi avaliada por goniometria. Para análise de adesão bacteriana, as amostras foram expostas durante 72 horas a uma solução de *Streptococcus mutans,* para formação de biofilme. Os valores obtidos para a rugosidade da superfície foram analisados pelo teste de Mann-Whitney, enquanto a adesão do biofilme foi avaliada pelos testes de Kruskal-Wallis e SNK.

**Resultados:**

observaram-se diferenças estatisticamente significativas (*p* <0,05) para a rugosidade superficial e redução da adesão bacteriana em braquetes convencionais após o tratamento da superfície, e entre braquetes convencionais e autoligáveis. Não foram observadas diferenças estatísticas significativas entre os grupos autoligáveis (*p*> 0,05).

**Conclusão:**

a deposição de polímero a plasma só foi efetiva na redução da rugosidade superficial e adesão bacteriana em braquetes convencionais. Observou-se, também, que os braquetes convencionais apresentaram menor adesão ao biofilme do que os braquetes autoligáveis, apesar da ausência de filme.

## INTRODUCTION

The amount of sites available to bacterial growth in oral cavity increases in the presence of orthodontic appliances. Therefore, surfaces traditionally unlikely to develop caries become areas with high incidence of these lesions.^1^ In the absence of prophylactic measures, initial carious lesions (white spots) develop within four weeks.^2^ Caries have a multifactorial trait, being dependent on the presence of the host (teeth), diet (sugars intake), cariogenic bacteria (biofilm) and the biofilm’s development stage, to be sustained. Thus, the absence of one of these factors may inhibit disease installation and its development.^3^

*Streptococcus mutans* is one of the main responsible microorganisms for tooth decay. Its installation is dependent on vertical and/or horizontal contamination and it has acidogenic and acidophilic characteristics^4^. Their carbohydrate degradation metabolism produces acids that demineralize dental surfaces, leading to the development of cavities.^5^

Bacterial adhesion has special characteristics and depends on direct biofilm interaction with the substrate surface to which it relates. The presence of acquired enamel pellicle,^6^ surface energy,^7^ roughness,^8^ and wettability^9^ play critical roles in this interaction, not only interfering in adhesion properties, but also in the characteristics of biofilm formation. 

The recent use of self-ligating brackets in orthodontics has contributed for a reduction in plaque accumulation, when compared to conventional brackets ligated by elastics,^10^ but the performance of these brackets may be impaired by salivary calculus accumulation over the sliding clip mechanism and into the horizontal archwire slot.^11^

Plasma polymerization and plasma surface treatment techniques have been developed as antibacterial coatings, such as silver-platinum coating for orthodontic appliances^12^ and TiO_2_ nanotubes surfaces coated with magnetron-sputtered Ag, for dental applications.^13^ Recent literature reports strategies in which plasma polymers have also been used as reservoirs loaded with antibacterial agents which are subsequently released,^14^ ^-^ ^17^ served as a diffusion barrier to control the release rate of these agents - as sealing agents for carbon nanotubes filled with medication,^18^ and as functional coatings for connecting antibiotic or bacteriostatic molecules.^19^

This practice, quite common in implantology^20^ serves as inspiration for orthodontics, where polymer films deposition on orthodontic brackets surface can also be applied to reduce biofilm adhesion and the risk of enamel lesions during treatment.^21^

Due to its characteristics of producing nontoxic films,^22^ having high vapor pressure at room temperature and being of ease commercial availability,^23^ the plasma-polymerized hexamethyldisiloxane (HMDSO) deposition has been largely employed in industry^24^ and as biomaterial coating^25^. HMDSO film presents several organic components and large hydrophobicity.^26^ These characteristics have particular importance in inhibiting the adherence of *Streptococcus mutans*.^27^

The objective of the present study was to compare the performance of the HMDSO film as a surface roughness reduction method and as an inhibiting barrier for biofilm formation in two kinds of orthodontic brackets, comparing its efficiency with non-treated brackets. The null-hypothesis was that the presence of HMDSO film would not interfere on biofilm formation on the two kind of brackets.

## MATERIAL AND METHODS

The study was composed by two groups (n = 34), equally divided by type of bracket, and in two subgroups, according to film deposition (Table 1).

**Table 1:** Groups division and names according to surface treatment and bracket type.

Group	Sample
SW	Self-ligating brackets with HMDSO polymer deposition
SO	Self-ligating brackets without HMDSO polymer deposition
CW	Conventional brackets with HMDSO polymer deposition
CO	Conventional brackets without HMDSO polymer deposition

For the HMDSO deposition and subsequent microbiological tests, the following upper right central incisors metallic brackets (Roth prescription, Morelli™, Sorocaba/SP, Brazil) were used: SLI (self-ligating) and Roth Max (conventional), both types manufactured by powder injection molding (PIM). The chemical composition of brackets used was C = 0.20% (max), Cr = 16.5 - 17.5%, Mo = 3.0 - 3.5%, Si = 1.0% (max) and Ni = 0.90% (max). Self-ligating brackets also presented a sliding clip composed by Ni = 54.5 - 57.0%W and Ti = 45.5 - 43.0%W, which plays an interactive hole in this bracket system. 

Each subgroup was composed by 17 samples, in which 2 were chosen to undergo scanning electron microscopy (SEM, Zeiss, model EVO LS15), 5 were used for confocal interferometry (CI) (Leica, DCM3D) and 10 for biofim formation analysis.

Scanning Electron Microscopy (SEM) was used to visualize the surface after polymer deposition. For assessing bacterial adhesion presence over samples surface (slot and wings areas) and back-scattered electrons (BSE) mode assessment of the film layer integrity over the samples, self-ligating brackets had their clips opened. CI was carried out to evaluated surface roughness (Ra, arithmetic average, and Rq, root mean squared) on the wings region. Prior to SEM and CI analysis, 7 brackets of each group were fixed for one hour in 2.5% glutaraldehyde, and dehydrated at various concentrations of ethanol (10%, 25%, 50%, 75% and 90% for 20 min, and 100% for 1 hour). To complete samples drying, they were incubated in a bacteriological incubator for 48 h at 37^o^C. 

### HMDSO films deposition system

Film deposition was performed by Plasma-Enhanced Chemical Vapor Deposition radio frequency method (RF PECVD), using the hexamethyldisiloxane (HMDSO) monomer as gas source. HMDSO gas plasma was RF-excited, operating in a 13.56 Hz frequency, and pressure level of 60 x 10^-2^Torr, with 20 W power, for 15 minutes. These parameters were chosen as the most adequate by a series of previous tests performed by the authors with different power and time periods. The film was deposited on the outer and inner surfaces of the brackets, while their bases remained facing the surface of the deposition electrode plate on the bottom of the reactor. All self-ligating brackets had their clips closed during the deposition process.

An automated goniometer (Ramé-Hard Instrument Co., Advanced Goniometer model 300-F1) was used for evaluating the wettability and surface energy on a stainless steel sheet presenting the same chemical composition of the brackets.

The thickness of HMDSO film was measured in an optical microscope and interferometer (Leica, DCM3D) on a glass slide substrate,^28^ which was set inside the plasma reactor amongst the samples during the deposition process and prepared to present a step on the surface between the film and the substrate.

### Biofilm formation

*Streptococcus mutans*, ATCC #35688 strains were used for biofilm formation, as proposed by Pereira et al.^29^ Initially, the strains were seeded in Mitis Salivarius to verify its purity, and incubated at 37^o^C for 24 hours. Standardized suspensions were then prepared with relative optical density at 10^6^ cells/mL. For this, the strains were grown on brain heart infusion agar (BHI, Difco, Detroit, USA) and incubated at 37^o^C for 24 hours. After incubation, the growth was suspended in sterile saline (0.9% NaCl) and the number of cells in each suspension calculated in a spectrophotometer (B582, Micronal, São Paulo, Brazil). Each bracket group was placed in a 12-well plate (Costar Corning, New York, USA) with 1.5 ml BHI plus 5% sucrose, and inoculated with 0.1 mL of bacterial suspension. The samples were incubated at 37^o^C for 72 hours for the formation of biofilms. After this period, the brackets with biofilms were rinsed with phosphate-buffered saline (PBS) and subjected to an orbital shaker for 5 minutes (Solab, Piracicaba, Brazil) for removing non-adhered cells. After proper dilutions, 100-µL aliquots were plated on BHI agar in Petri dishes. The plates were incubated at 37^o^C for 72 hours. After that period of incubation, the colony forming units per milliliter (CFU/mL) were quantified on the plates showing from 30 to 300 colonies, and the obtained numbers were converted to their corresponding logarithm (log_10_ CFU/mL). Statistical analysis was performed with Sigmastat v. 4.0 software (Systat Software Inc., San Jose, USA).

The Kolmogorov-Smirnov test was carried out to analyze data normal distribution of four groups considering the following assumptions:

» Null hypothesis (H0) = Analyzed data distribution is similar to a standard normal distribution.
» Alternative hypothesis (Ha) = Analyzed data distribution is not similar to a standard normal distribution.

The Mann-Whitney (surface roughness between subgroups) and Kruskal-Wallis (bacterial adhesion) tests assessed possible differences among groups, considering the following hypotheses:

» Null hypothesis (H0) = Analyzed data are similar among the groups.
» Alternative hypothesis (Ha) = There is at least one group different from other groups.

To identify all the possible differences among groups, SNK multiple comparison test was applied. 

## RESULTS 

**Figure 1:**
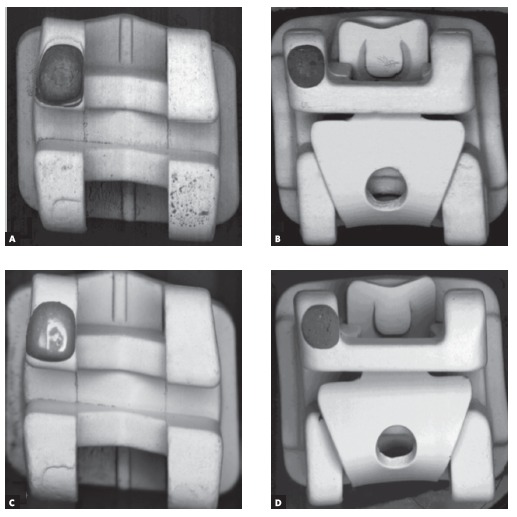
Top view of the brackets surfaces: A, B) non-treated samples, C, D) treated samples (45x magnification).

Micrographs of two groups of brackets in BSE mode can be observed in Figure 1, where differences in the atomic number of the surface layer molecules create contrast variations, highlighting possible deposition defects. Surface visual analysis of the four brackets subgroups - self-ligating and conventional, treated and not treated - exhibited a uniform layer deposition pattern on treated samples after polymer deposition.

**Figure 2:**
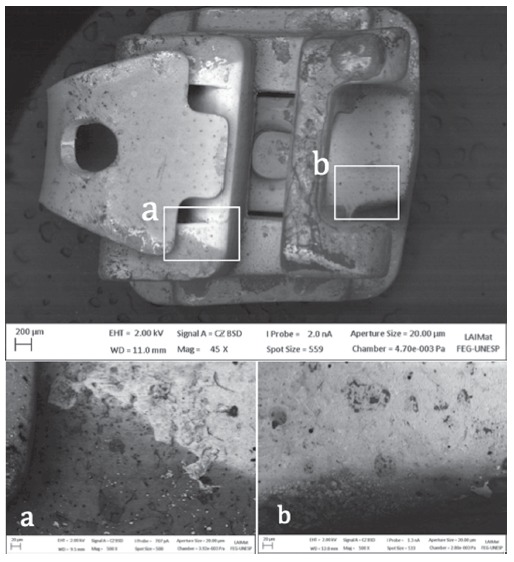
SEM micrograph shows the projected shadow impressed by the presence of the NiTi clip, creating an interface between the surfaces with deposition and non-deposition (45x magnification). Interface on the wing region (a) and on bracket base region (b) (500x magnification).

A fairly uniform layer was observed at samples C and D compared to the conditions on untreated samples A and B (Fig. 1). This evidences the proper deposition of the polymer over the outer surface of the brackets. Despite that, SEM analysis of a SW group bracket with its clip open had shown the presence of surface areas without proper coating (Fig 2). 

Surface roughness(Ra and Rq) median values of the wings region of the samples are presented in Table 2.

**Table 2:** Confocal Interferometry (CI) evaluation of surface roughness (Ra and Rq).

Group	Ra (median)	Rq (median)
CO	3.760	4.963
CW	1.623	2.192
SO	1.749	2.296
SW	1.649	2.177
(*p* < 0.05)		

Data analysis by Mann-Whitney test has shown no significant statistical differences on surface roughness reduction between subgroups SW and SO for Ra (*p*= 0.222) and Rq (*p*= 0.151). Significant statistical surface roughness reduction for Ra (*p*= 0.008) and Rq (*p*= 0.008) was observed between subgroups CW and CO. 

The HMDSO film wettability and surface energy analysis performed with the goniometer, on a stainless steel sheet, had shown that polymerized samples presented hydrophobic surface characteristics, while control group presented hydrophilic characteristics to deionized water (polar) and to diiodomethane (apolar), as shown in Table 3. This result shows that surface polymer characteristics were present after polymerization.

**Table 3:** Angles and surface energy measurement observed by the goniometer on the different samples.

Sample	Contact angle				
	Water	Diiodomethane	Polar component	Dispersive component	Total surface energy
Control	79.22 ± 4.43	48.45 ± 0.45	9.49 ± 1.90	36.16 ± 0.22	45.65 ± 1.91
Deposition 1	105.78 ± 1.08	81.67 ± 1.51	2.87 ± 0.44	20.37 ± 0.67	23.24 ± 0.60
Deposition 2	99.87 ± 0.22	72.67 ± 0.47	3.84 ± 0.10	24.40 ± 0.22	28.24 ± 0.18
Deposition 3	103.42 ± 1.15	83.36 ± 0.80	3.92 ± 0.42	19.65 ± 0.34	23.57 ± 0.46

The presence of the polymer in an uniform layer, with regular thickness, was assessed by the interferometry of the coated layer film thickness on the glass slide, after the deposition of plasma-polymerized HMDSO (Table 4).

**Table 4:** Film thickness obtained from plasma deposition parameters.

Sample	Film thickness (nm)
1	10.36
2	11.3
3	11.25
Mean	10.97

**Figure 3:**
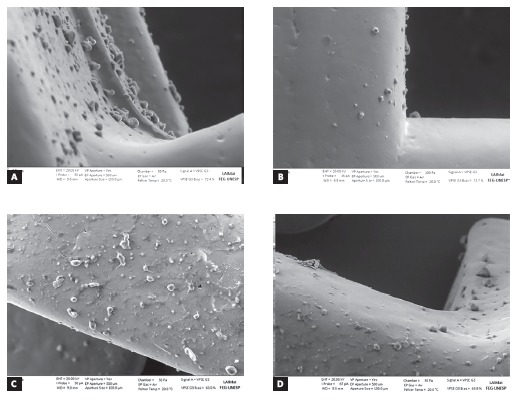
SEM micrographs of the brackets slots (A, B) and wings region (C, D) demonstrate the bacterial adhesion in both regions of all groups (4,500x magnification).

Micrographs observed in Figure 3 show the worst areas of *S. mutans* biofilm formation on all subgroups, on the slots (Figs 3A and 3B, lateral view) and wings regions (Figs 3C and 3D, frontal view). In SW (Fig 3A) and SO (Fig 3C) groups a greater bacterial adherence was observed, both in the wings and slot regions, when visually compared to CW (Fig 3B) and CO (Fig 3D) groups, even in the presence of the HMDSO film (Fig 3).

Descriptive statistics of the colony forming units (CFU) data can be observed in Table 5. 

**Table 5:** Descriptive statistics: mean, standard deviation (SD), median, first quartile (Q1), third quartile (Q3), minimum and maximum values, for the values of colony counts (log).

Group	Mean (S.D.)	Median	Q1	Q3	Min	Max
SO	9.13 (0.63)	9.06^A^	8.60	9.47	8.45	10.28
SW	9.00 (0.31)	8.93^A^	8.78	9.05	8.76	9.85
CO	7.99 (1.82)	8.50^B^	8.01	8.77	3.00	9.48
CW	5.79 (2.78)	6.99^C^	3.78	7.59	0.00	8.92

The results of Kolmogorov-Smirnov tests identified that SO and CW groups showed normal distribution (*p*> 0.05) and that SW and CO groups were not normally distributed (*p*< 0.05), whereas the level of 5% significance was adopted, so non-parametric statistical analyzes were chosen to analyze the data. 

The result of the Kruskal-Wallis test indicated the presence of at least one group different from the other groups (H = 18.56, *p* < 0.001). SNK multiple comparison test indicated greater accumulation of bacteria in groups SO and SW, and smaller accumulation in CW group (*p*< 0.05) (Table 5). 

## DISCUSSION

HMDSO polymer films have been largely used as surface treatment for biomaterials^25^ and in dentistry as carriers for drug delivering^16^ and for surface wettability modification.^22^ This paper used the HMDSO polymer to observe its anti-adherence features on biofilm formation, benefiting from its hydrophobicity characteristics^26^ and layer thickness, without any associated anti-bacterial substance, once in orthodontics, brackets remain in oral cavity for a period of time that varies from 12 to 30 months and most drug delivering properties occur in a brief period of time, varying from 24 to 48 hours. Surface properties play an essential hole in bacterial adhesion: hidrophobic and high surface energy types of surface, as the ones observed in the treated samples, tend to difficult such interaction.

RF-PECVD technique was chosen for its capability of controlling film thickness and wettability characteristic according to determined deposition parameters, producing uniform and very thin films. Film thickness measurement was facilitated by its deposition on a glass slide surface,^28^ once bracket surface irregularities made interferometry assessment impracticable. SEM analysis on BSE mode have shown a uniform polymer surface deposition. The deposition process of HMDSO polymer allows samples coating in all dimensions of space, and assures high rates of deposition.^26^ Despite that, the base of the brackets did not receive any coating, once it was facing down the base of the reactor. 

The base of the bracket itself had little influence on the amount of adhered biofilm on total bracket surface, once most of the brackets surface and its harsh design are determined by its outer surface and not by the bracket base. 

Brackets brand and type choice was based on their composition and manufacturing process, and the same brand was chosen for both brackets types; only brackets geometry and presence of the NiTi clip varied in the self-ligation groups. 

Literature review^10^ ^,^ ^30^ demonstrated that conventional brackets have shown less plaque buildup than self-ligating brackets. Besides that, self-ligating brackets as well as ceramic brackets provide the formation of a much more pathogenic biofilm, due to their small proportion of anaerobic over aerobic bacteria in colony forming units (CFU).^30^ Therefore methods for reducing plaque adherence, especially on self-ligating brackets are very welcome.

Roughness tests were performed only between subgroups once the main concern was the influence of the presence of polimer coating. The low influence of polymer deposition on biofilm formation in SW and SO groups was directly related to the small reduction on surface roughness and highly detailed and complex geometry of this type of bracket, which had a large influence on bacterial adherence.^10^ Surface roughness influence overrules surface free energy and promotes plaque formation and maturation.^7^

The presence of a NiTi clip as the ligation element in the bracket implies in the presence of an internal longitudinal tunnel, just above the base of the bracket, for accommodating the NiTi clip. This feature creates a broad contact surface in this type of bracket and results in a perfect site for bacterial adherence and proliferation. In the conventional CO and CW groups, which have a much simpler geometry, this tunnel is not present.

This characteristic, in addition to the untreated surface created by the clip shadow, resulted in more bacterial adherence on SW group than on CO. Even in the absence of the polymer coating, CO group has shown lower rates of bacterial adhesion than SO and SW groups, thus demonstrating that the impact of the external geometry and polymer deposition flaws on biofilm formation was bigger than the presence of the polymer. 

HMDSO film deposition on group SW had serious issues, as shown in Figure 2. This experiment was held with the NiTi clip closed, ​​following the same methods conducted by other authors on their experiments on bacterial adhesion.^10^ ^,^ ^30^ ^-^ ^32^ This option was made because the removal of the clip for film deposition would imply its repositioning, what could allow the incorporation of grooves and imperfections on brackets surfaces and negatively interfere on bacterial adhesion. A second deposition round performed with the clips open could have eliminated the shadow areas were film was not present, but this would modify film characteristics due to the risk of sputtering of the initial layer.^25^ Future experiments should perform film deposition on self-ligating brackets with the clip open, or without the clip, to observe possible improvement in polymer deposition on inner surfaces of the samples.

Findings have shown that CW group had the best results on reducing bacterial adherence over all groups, demonstrating that the polymer film played a fundamental role in reducing surface roughness and the rate of bacterial adhesion. This very positive outcome unravels a new perspective to surface treatment in order to reduce bacterial adhesion in orthodontics, and set HMDSO polymer as a feasible choice for metallic orthodontic brackets coating.

This benefit, not yet commercially available, was also verified by Demling et al,^21^ who conducted an *in vivo* study comparing bacterial adhesion in two conventional brackets, one of them coated with plasma-polymerized polytetrafluoroethylene. Despite being presented as a case report, the authors observed a much smaller amount of bacteria adhered to the surface of brackets with film deposition (4.0 ± 3.6%), compared to brackets without film (22.2 ± 5.4%). This primer study can serve as a reference for the indication of plasma surface treatment of brackets in orthodontics.

For the special characteristics observed, the authors acknowledge the necessity of improvement in self-ligating brackets polymer deposition method, as well as the use of different bracket brands, with different external geometries and ligation features. The development of a bacterial adherence inhibiting method is essential for this kind of bracket, once biofilm presence, besides enamel lesions, can also interfere in the opening mechanism of the clip and in the proper interaction between the bracket and the archwire, leading to mechanical and operational problems during the orthodontic treatment. 

Besides this, further studies shall be performed regarding possible friction reduction between treated brackets and different archwires, due to the surface roughness reduction observed in polymer deposition groups. 

## CONCLUSIONS

The results observed in this paper allow the following conclusions concerning the HMDSO polymer deposition on orthodontic brackets:

» It was more effective in reducing surface roughness and *S. mutans* biofilm formation in conventional brackets, for their less rugged and more suitable external geometry, which enabled a better polymer film deposition.
» Conventional brackets showed lower biofilm adhesion than self-ligating brackets despite the absence of film.
» An improved deposition method has to be employed in self-ligating brackets so that film deposition and hence, the reduction in bacterial adhesion and surface roughness, may be more effective.
